# Fabrication of 3D printed head phantom using plaster mixed with polylactic acid powder for patient-specific QA in intensity-modulated radiotherapy

**DOI:** 10.1038/s41598-022-22520-6

**Published:** 2022-10-19

**Authors:** Sung Yeop Kim, Jae Won Park, Jaehyeon Park, Ji Woon Yea, Se An Oh

**Affiliations:** 1 Department of Physics, Yeungnam University, Gyeongsan, Korea; 2 Department of Radiation Oncology, Yeungnam University Medical Center, Daegu, Korea; 3 Department of Radiation Oncology, Yeungnam University College of Medicine, 170, Hyeonchung-Ro, Nam-Gu, Daegu, 705-717 South Korea

**Keywords:** Cancer therapy, Radiotherapy

## Abstract

This study aimed to fabricate a heterogeneous phantom replicating the commercial Rando phantom by mixing plaster powder and polylactic acid (PLA) powder. Producing a heterogeneous phantom using Plaster and PLA is cheaper because it can be easily obtained in the commercial market. Additionally, patient-specific Quality Assurance can be easily performed because the phantom can be produced based on the patient’s CT image. PLA has been well studied in the field of radiation therapy and was found to be safe and effective. To match the mean Hounsfield unit (HU) values of the Rando phantom, the bone tissue was changed using plaster and 0–35% PLA powder until an appropriate HU value was obtained, and soft tissue was changed using the PLA infill value until an appropriate HU value was obtained. Bone tissue (200 HU or higher), soft issue (− 500 to 200 HU), and air cavity (less than − 500 HU) were modeled based on the HU values on the computed tomography (CT) image. The bone tissue was modeled as a cavity, and after three-dimensional (3D) printing, a solution containing a mixture of plaster and PLA powder was poured. To evaluate the bone implementation of the phantom obtained by the mixture of plaster and PLA powder, the HU profile of the CT images of the 3D-printed phantom using only PLA and the Rando phantom printed using only PLA was evaluated. The mean HU value for soft tissue in the Rando phantom (− 22.5 HU) showed the greatest similarity to the result obtained with an infill value of 82% (− 20 HU). The mean HU value for bone tissue (669 HU) showed the greatest similarity to the value obtained with 15% PLA powder (680 HU). Thus, for the phantom composed of plaster mixed with PLA powder, soft tissue was fabricated using a 3D printer with an infill value of 82%, and bone tissue was fabricated with a mixture containing 15% PLA powder. In the HU profile, this phantom showed a mean difference of 61 HU for soft tissue and 109 HU for bone tissue in comparison with the Rando phantom. The ratio of PLA powder and plaster can be adjusted to achieve an HU value similar to bone tissue. A simple combination of PLA powder and plaster enabled the creation of a custom phantom that showed similarities to the Rando phantom in both soft tissue and bone tissue.

## Introduction

Effective radiation therapy requires delivery of high doses of radiation to the tumor and minimal doses to the surrounding normal tissues^1,2^. In particular, optimization of the dose distribution in intensity-modulated radiotherapy (IMRT), volumetric-modulated arc therapy (VMAT), and stereotactic body radiation therapy (SBRT) is a complex procedure, and treatment beams do not uniformly deliver optimal quantities of radiation to patients^3–5^. Therefore, an important step in the process is to verify the planned dose distribution using the radiation treatment planning system (RTPS). Two major methods are used in this regard: evaluations using independent secondary dose calculation software, and measurements using chamber, film, or diode-array detectors^3,6^. However, for patient-specific quality assurance (QA), independent secondary dose calculation software shows some errors in confirming the actual radiation dose distribution^4,7^. Therefore, measurements using detectors are recommended for patient-specific QA.

Advancements in three-dimensional (3D) printers have facilitated the production of bolus, compensators, and anthropomorphic phantoms with geometrically elaborate and customizable properties^1,5,7–9^. Park et al.^8^ used a 3D printer to fabricate a bolus that was able to reduce the air gaps caused by nose flexion. In a study by Zou et al.^9^, they used a 3D printer to produce a compensator that could accommodate irregularities, tissue inhomogeneity, and planning target volume (PTV) depth changes in the patient's body surface to achieve the expected dose distribution. There are currently numerous studies being conducted on the fabricate of anthropomorphic phantoms for patient-specific QA through measurements^1,5,7^. Kamomae et al. and Yea et al.^1,7^ fabricated an anthropomorphic phantom using a 3D printer for patient-specific QA. However, because of the low Hounsfield unit (HU) values of acrylonitrile butadiene styrene (ABS) and polylactic acid (PLA) filaments, the differences in HU values between bone and soft tissue cannot be expressed^1,7^. Several existing papers have reported on phantoms produced by expressing the HU difference between bone and soft tissue^10,11^. Kadoya et al.^10^ produced a head phantom using plaster to express bone tissue. However, the average HU of bone tissue could not be properly replicated. In addition, the HU value of the plaster and the change in the HU value over time of the plaster could not be adjusted. Ali et al.^11^ produced a phantom using plaster for the ceramic bone of the pelvis. In addition, by adjusting the ratio of plaster and water, the change in the HU value and the change in the HU value of plaster over time were shown. However, according to Li et al.^12^, as the ratio of water increases, pores are formed in the plaster.

In this paper, the change in HU value and the change in HU value over time, according to the change in the ratio of PLA powder and plaster, were investigated. In this way, our study aimed to determine the PLA powder percentage and infill value suitable for reproducing the mean bone and soft tissue HU values of the commercial Rando phantom. In addition, we also compared the PLA powder/plaster phantom prepared using the appropriate PLA powder percentage and infill value with a 3D-printed phantom made using only PLA filaments.

## Materials

### Workflow overview for phantom fabrication composed of plaster mixed with PLA powder

**Figure 1:**
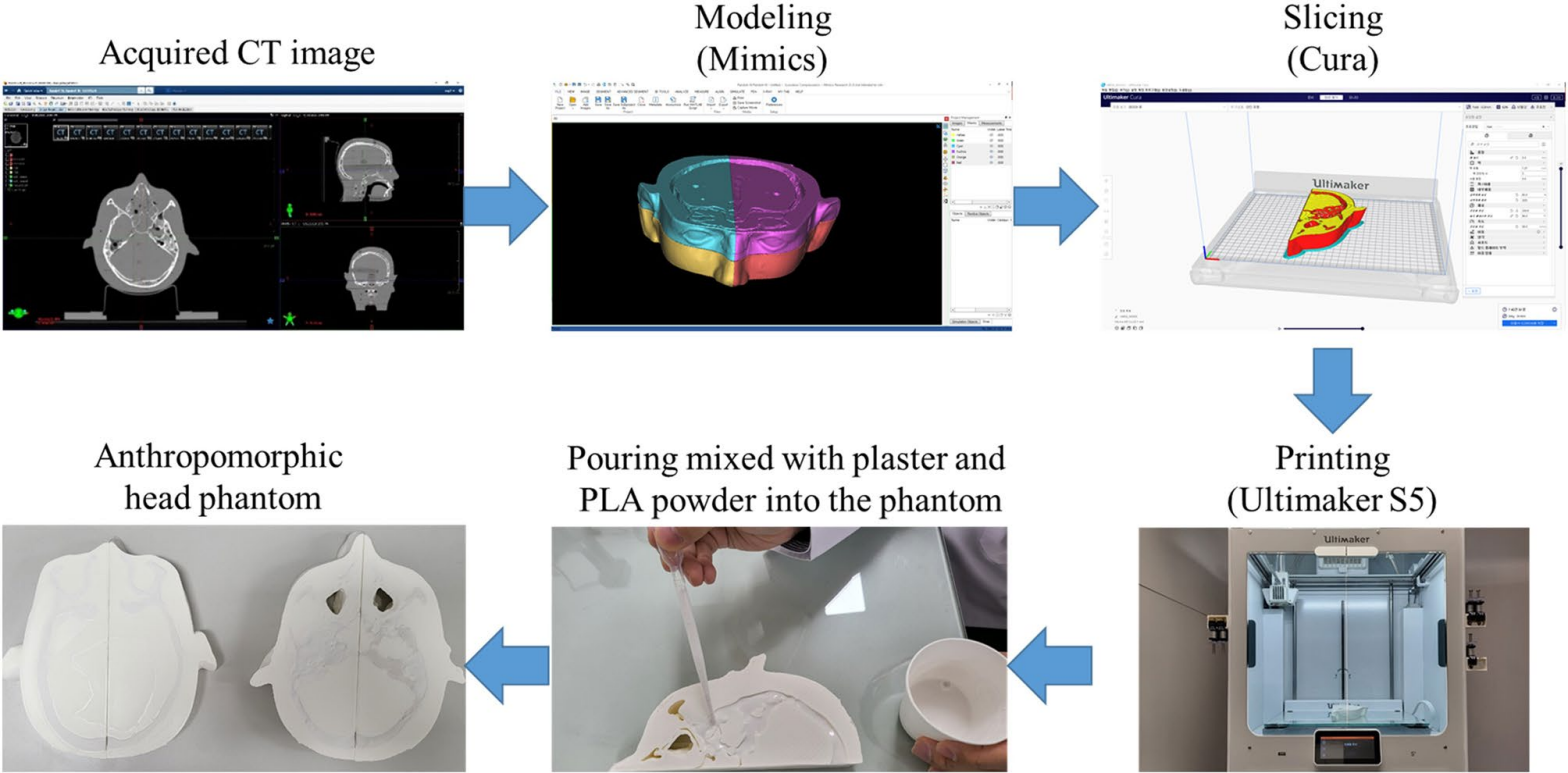
Workflow for preparing a phantom composed of plaster mixed with PLA powder.

Figure 1 presents a workflow for creating a phantom composed of plaster mixed with PLA powder. The production process of this phantom encompassed six stages. The first step involved a CT scan of the head of an Alderson Rando phantom (The Phantom Laboratory, Salem, NY, USA). All CT scans were conducted used the Philips Big Bore Brilliance CT Scanner (Philips Medical, Eindhoven, Netherlands) at an X-ray tube voltage and current of 120 kV and 125 mA, respectively, and a slice thickness of 1 mm. Second, the scans were imported into Mimics 21 software (Materialise, Leuven, Belgium) using Digital Imaging and Communication in Medicine (DICOM) files. Based on the voxel HU value of the CT image, the threshold function was used to model only the voxel having the HU value in the area desired by the user. The software was used to model three areas (air cavity, bone, and soft tissue) on the basis of HU values. Soft tissue was represented by an HU range of − 500 to 200 HU, while bone was represented by HU values greater than 200 HU^7,10^. The air cavity was set to − 500 HU or less. We modeled the bone tissue as a cavity. The modeling area only needs to consist of the area needed for patient-specific QA. Thus, we modeled only the region where the cavity, bone and soft tissue regions properly exist in the head phantom region. Third, the 3D model was exported to stereolithography (STL) format and then imported into CURA (Ultimaker, Utrecht, Netherlands), a slicer software for 3D printing. Fourth, the soft tissue was printed using a 3D printer to show an HU value similar to that of the Rando phantom through adjustment of the infill value. Fifth, the bone tissue was adjusted by modifying the ratio of plaster and PLA powder to show an HU value similar to that of the Rando phantom. After printing the bone tissue in the cavity, the liquid mixture of plaster and PLA powder was poured. Finally, the liquid mixture of plaster and PLA powder corresponding to the bone tissue was dried.

### 3D printer setup conditions

All phantoms were printed in a line pattern using white PLA (Ultimaker; Utrecht, Netherlands) with a density of 1.24 g/cm^3^, plaster (Heepani Tools, Pasadena, California), and the Ultimaker S5, using the fused deposition modeling (FDM) method. According to the manufacturer, the maximum build volume is 330 × 240 × 300 mm^3^ and the build speed is less than 24 mm^3^/s. The nozzle size was 0.8 mm with a nozzle operating temperature of 180–280 °C (https://ultimaker.com/3d-printers/ultimaker-s5). The settings of the 3D printer were as follows: layer height, 0.3 mm; shell thickness, 1.37 mm; printing speed, 50 mm/s; nozzle temperature, 230 °C; and bed temperature, 80 °C.

### Bone and soft tissue selection

To obtain soft tissue HU values similar to those of the Rando phantom, the infill values of the cuboid specimens (50 × 50 × 10 mm) were varied (5%, 20%, 50%, 70%, and 100%). Infill values, which were defined by Madamesila et al.^13^, represented the mean ratio of the printed thermoplastic volume (printed PLA area) to the air volume.

For bone tissue, the ratio of Orthodontic Gemma 24 plaster (Samwoo Co., Ltd., Seoul, Korea) and PLA powder (particle size: approximately 125 μm) was adjusted to yield HU values similar to those of the Rando phantom. The density of the plaster was 2.3 g/cm^3^. Table 1^14,15^ indicates the physical properties of plaster and PLA used in production. Table 2 shows the mixture ratios of plaster and PLA powder. The total mass of the PLA powder and plaster was 50 g, and the percentage of PLA powder increased by 5% increments from 0 to 35%. Additionally, 24 g of water was mixed into the sample.

**Table 1:** Physical properties of plaster and PLA.

Material	Density (g/cm^3^)	Molecular weight (g/mol)	Chemical formula	Relative electron density *ρ*_e_/*ρ*_e,water_	Chemical composition (percentage by mass)				
					H	C	O	Ca	Etc
Plaster	2.32^a^	145.15^a^	CaH_2_O_5_S	1.66^d^	2.3^b^	–	55.8^b^	23.3^b^	–
PLA	1.24^a^	72	(C_3_H_4_O_2_)_n_	1.13^d^	5.3^c^	51.9^c^	42.6^c^	–	0.2^c^

^a^Provided by each fabricator.

^b^Ref^14^.

^c^Ref^15^.

^d^Measured by CT.

**Table 2:** Mixture ratios of plaster and PLA powder.

PLA powder (g)	Plaster (g)	Percentage of PLA powder (%)	Water (g)
0.0	50.0	0	24
2.5	47.5	5	
5.0	45.0	10	
7.5	42.5	15	
10.0	40.0	20	
12.5	37.5	25	
15.0	35.0	30	
17.5	32.5	35	

## Results

### Cuboid specimens infill value calibration

**Figure 2:**
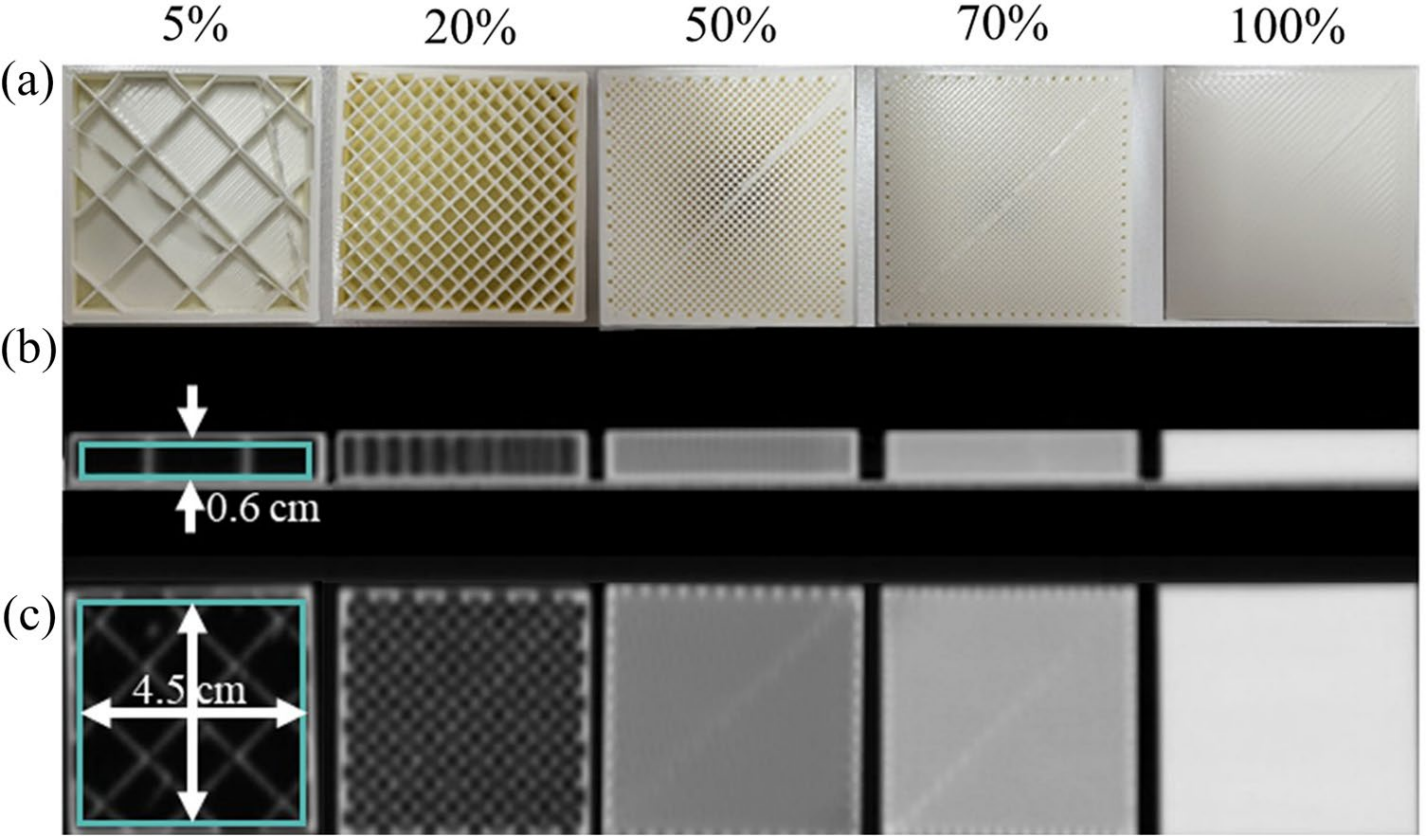
(**a**) Photograph of the cuboid specimens with infill values of 5%, 20%, 50%, 70%, and 100%. (**b**) Transversal and (**c**) frontal plane images of the cubic phantom with infill values obtained by CT scan.

Figure 2a presents a photograph obtained after removing the top wall to visualize the infill of the cuboid specimens. Figure 2b,c are CT images obtained with different infill values. A region of interest (ROI) was designated to check the mean HU value in the CT image. The ROI was 4.5 cm horizontal and 0.6 cm vertical on the transversal plane, as shown in Fig. 2b, and a 4.5 cm square on the frontal plane, as shown in Fig. 2c.

**Figure 3:**
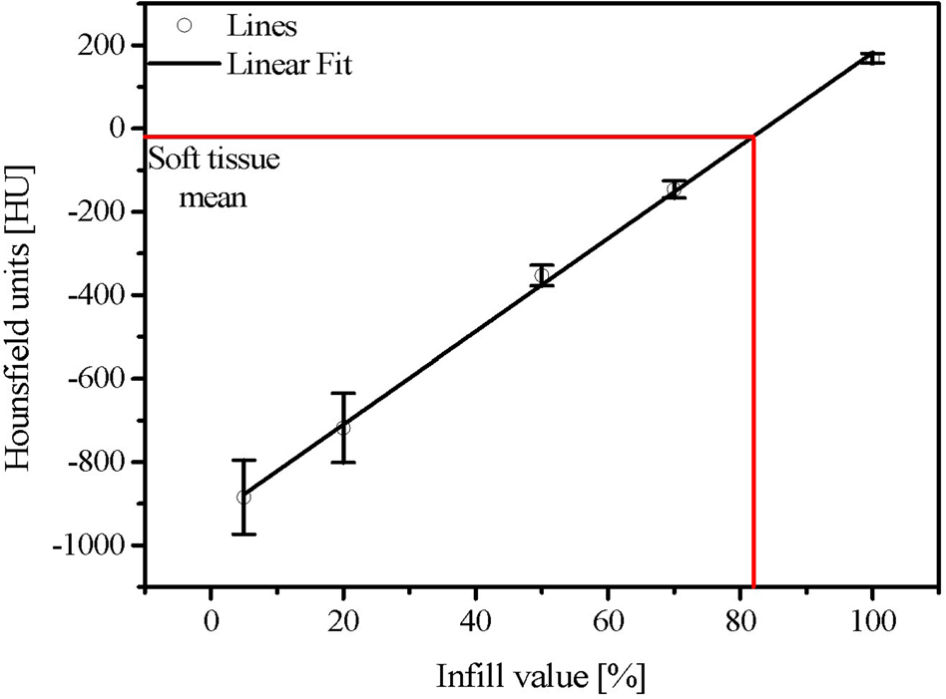
Mean HU values according to the infill value of the 3D printer.

Figure 3 shows the HU values according to the infill value. An infill value of 5% has a standard deviation of 88.8 HU. And an infill value of 100% has a standard deviation of 11.5 HU. As the infill value decreases, the value of the standard deviation decreases, meaning that the inside of the cuboid specimen becomes more uniform. Since the ratio of the printed thermoplastic volume and the air volume was altered to adjust the mean HU value with the 3D printer, the smaller the infill value, the more non-uniform it became. The Pearson’s correlation coefficient (r) was 0.999, confirming that the mean HU value showed a linear relationship with the infill value. The mean HU value was  indicates the physical properties of plaster 884.4 HU at an infill value of 5% and 169.0 HU at an infill value of 100%; the mean HU value increased as the infill value increased.

### PLA powder and plaster ratio

**Figure 4:**
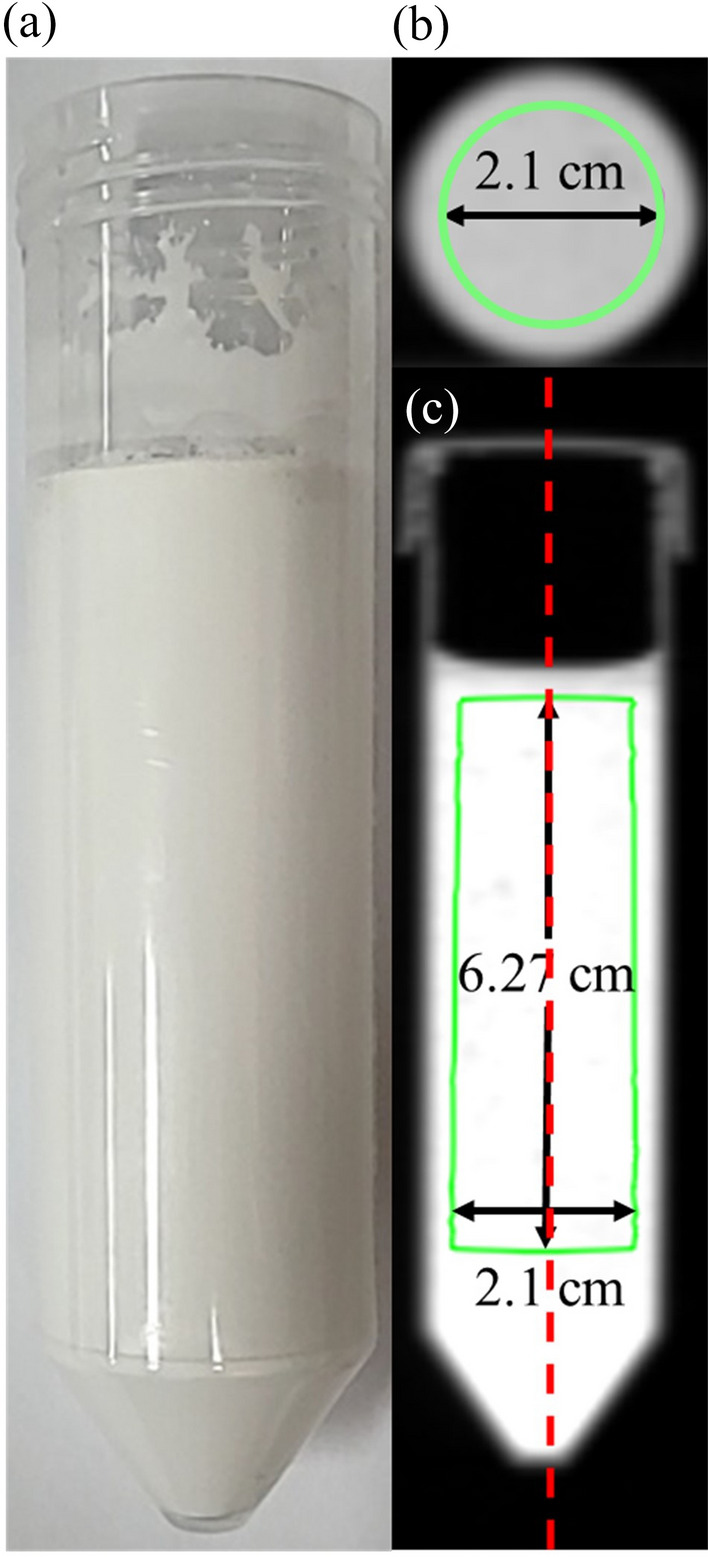
(**a**) Photograph of the mixture of plaster and PLA power. (**b**) Transversal and (**c**) frontal CT images of the samples.

Figure 4a presents a sample photograph of a conical tube with the mixture of plaster and PLA powder with water. The conical tube was stored in an open state to ensure that the mixture was in contact with the outside air at room temperature. Figures 4b,c show CT images of the mixture with 0% PLA powder. The ROI for measuring the mean HU value in Fig. 4b was selected as a circle with a diameter of 2.1 cm in the transversal plane and a height of 6.27 cm in the front plane in Fig. 4c.

**Figure 5:**
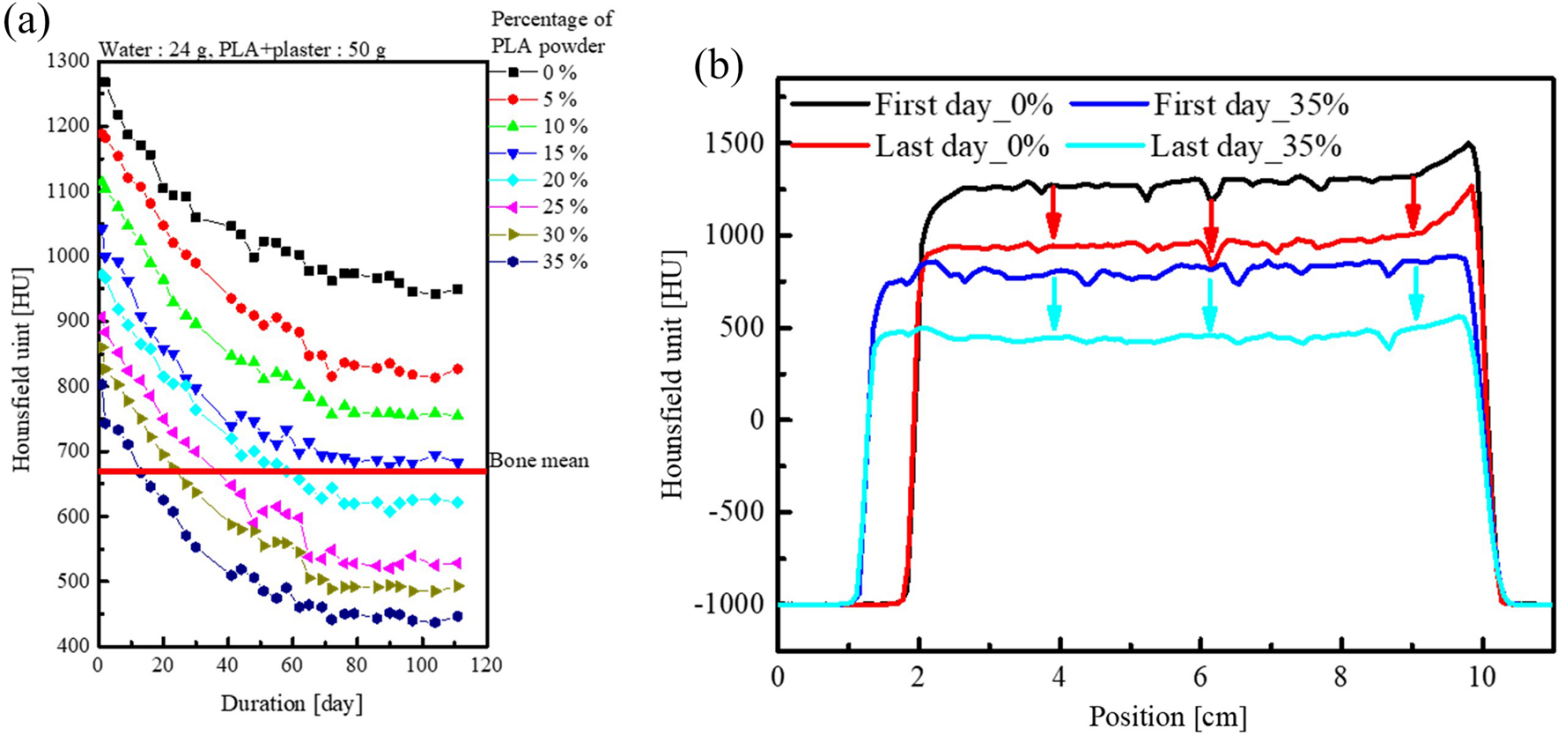
(**a**) Time changes in HU for the different percentage of PLA powder (**b**) HU profile of the red dotted line in Fig. 4c at 0% and 35%.

**Figure 6:**
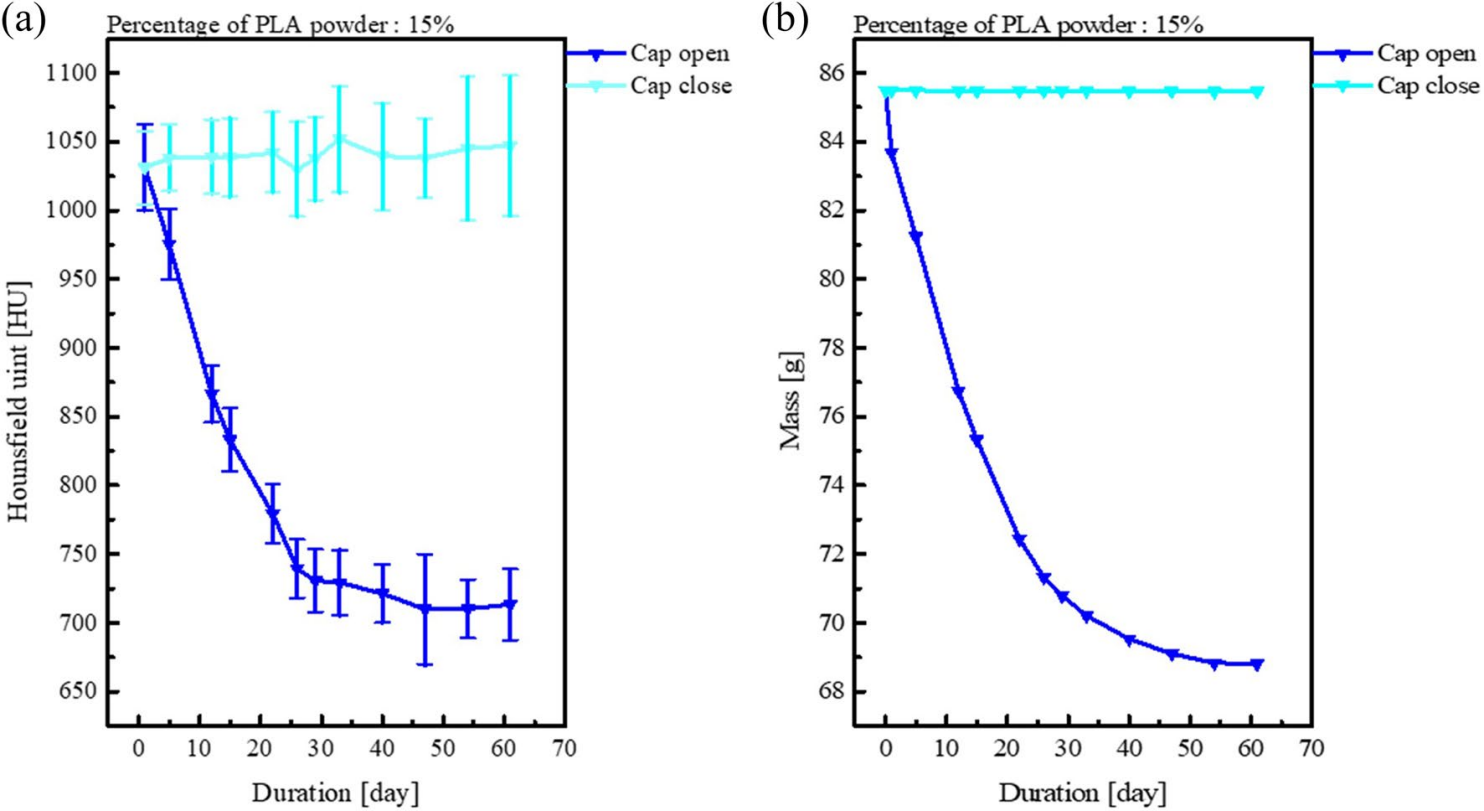
The differences in (**a**) HU and (**b**) mass over time with the conical tube open and closed.

Figure 5a shows the HU values over time for different percentages of PLA powder. The HU value decreased from 1269 to 803 HU as the percentage of PLA powder increased on the first day. For all PLA powder percentages, the HU values decreased by 319–379 HU over time. Figure 5b shows the sagittal direction HU profile of the fabrications shown in Fig. 4c for that of (c) 0 and 35 percent of PLA powder. It was confirmed that the HU value decreased regardless of the height. The decrease in the HU value was attributable to the evaporation of moisture from the open conical tube. Therefore, the mass and HU values were compared with the conical tube kept open and closed. Figure 6a compares the HU values in relation to the drying time in the open and closed conical tubes, while Fig. 6b presents the same comparison for mass. The samples in both open and closed tubes contained 15% PLA powder. For the closed condition, the mean HU value was between 1031 and 1052 HU, and the mass was 85.5 g, and the mean HU value and mass over time were almost constant. For the open condition, the mean HU value was between 1031 and 710 HU, and the mass was between 85.5 and 68.8 g, and the mean HU value and mass decreased over time.

### Determination of soft tissue and bone HU value for Rando phantom production

**Figure 7:**
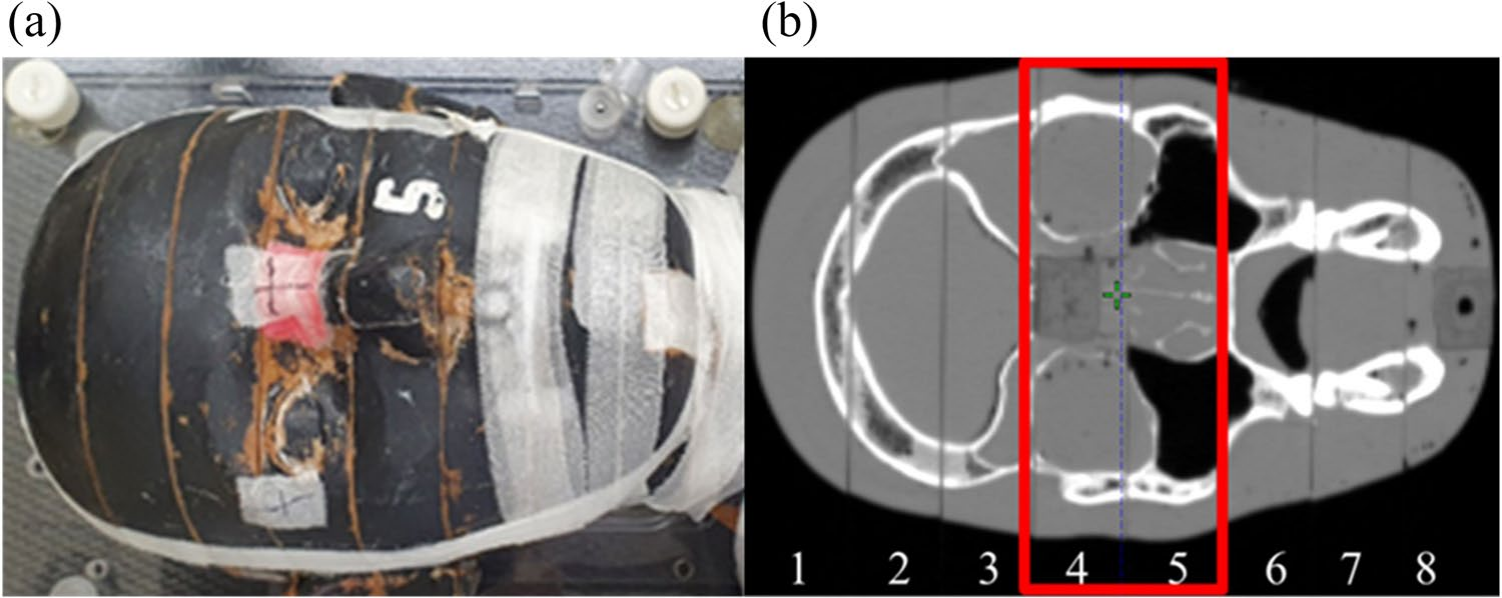
(**a**) Rando phantom and (**b**) CT image of the Rando phantom.

Figure 7 presents a photograph and CT image of the Rando phantom. Rando phantoms No. 4 and 5 shown in Fig. 7b, which clearly distinguished soft tissue, bone tissue, and air cavity, were implemented using PLA and plaster. The mean HU values of the Rando phantom were 669.2 HU for bone tissue and -22.5 HU for soft tissue. In Figs. 3 and 5, samples corresponding to the mean HU values of soft and bone tissue can be predicted. Soft tissue can be printed with a − 20 HU value by applying an infill value of 82% in Fig. 3. For bone tissue, the percentage of PLA powder, which showed the most similar HU value as feature 4 is 15%, which is expected to correspond to approximately 680 HU.

**Figure 8:**
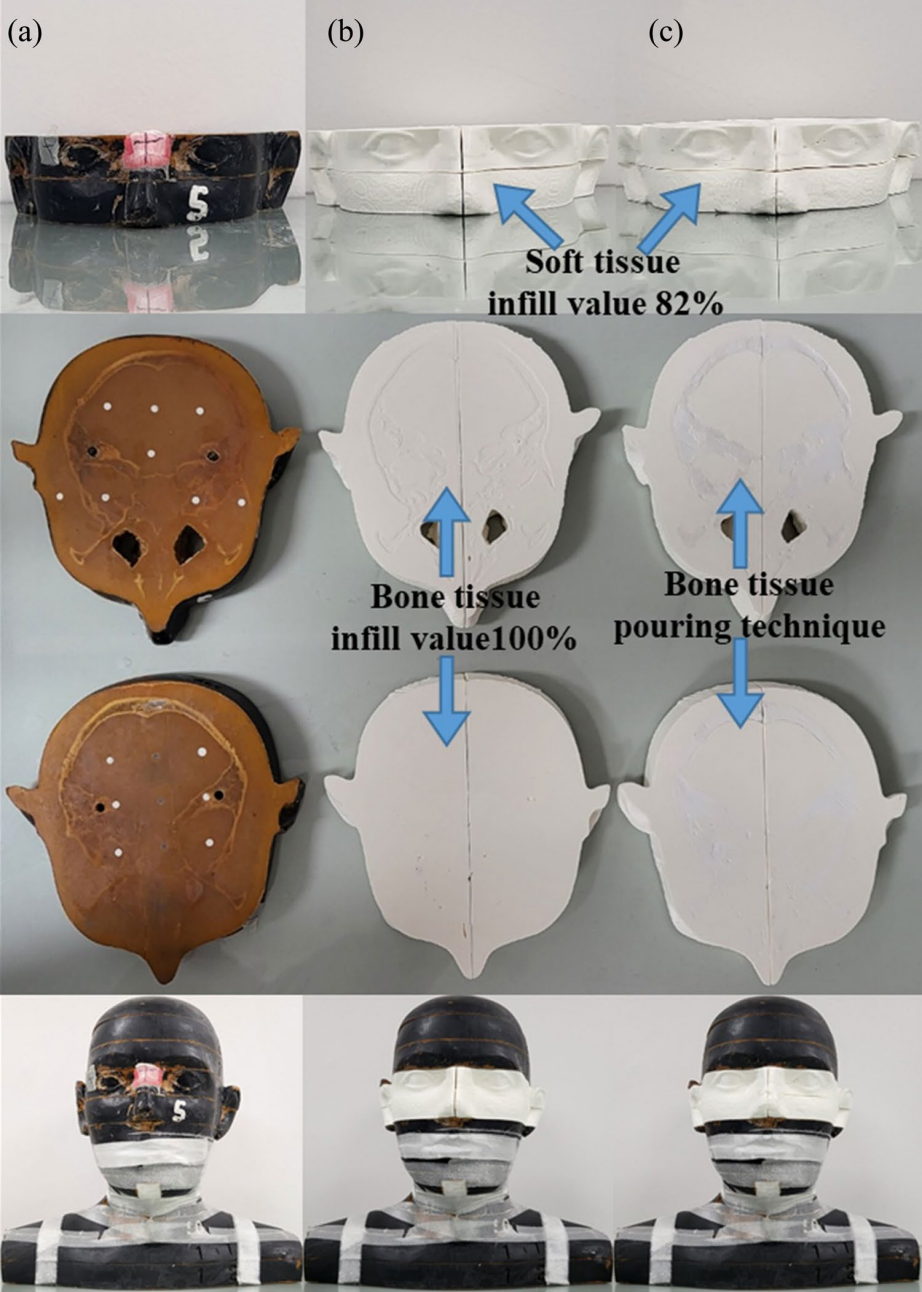
(**a**) Commercial Rando head phantom, (**b**) Rando head phantom printed using only PLA material, (**c**) Rando head phantom printed with a mixture of plaster and PLA powder.

After printing the bone tissue area into the cavity, the liquid mixture of the PLA powder and plaster was filled using a disposable dropping pipet. Figure 8 presents the phantoms fabricated using Rando phantom CT image and the Rando phantom. Figure 8b is a phantom fabricated using only PLA filament with a 3D printer. In the only-PLA 3D-printed phantom, the difference between bone and soft tissue was set by changing the infill value, with bone tissue generated using an infill value of 100% and soft tissue created using an infill value of 82%. Figure 8c shows the phantom fabricated using PLA filament, PLA powder, and plaster. The bone tissue region of the plaster mixed with PLA powder phantom also confirmed a decrease in HU due to evaporation of moisture and was dried until the HU became constant. Soft tissue was printed using an infill value of 82%. As shown in Fig. 1, bone tissue was implemented using PLA powder and pouring technique using plaster. In the phantom composed of plaster mixed with PLA powder, the mean HU value of bone tissue was 618 HU and the mean HU value of soft tissue was − 7.5 HU.

**Figure 9:**
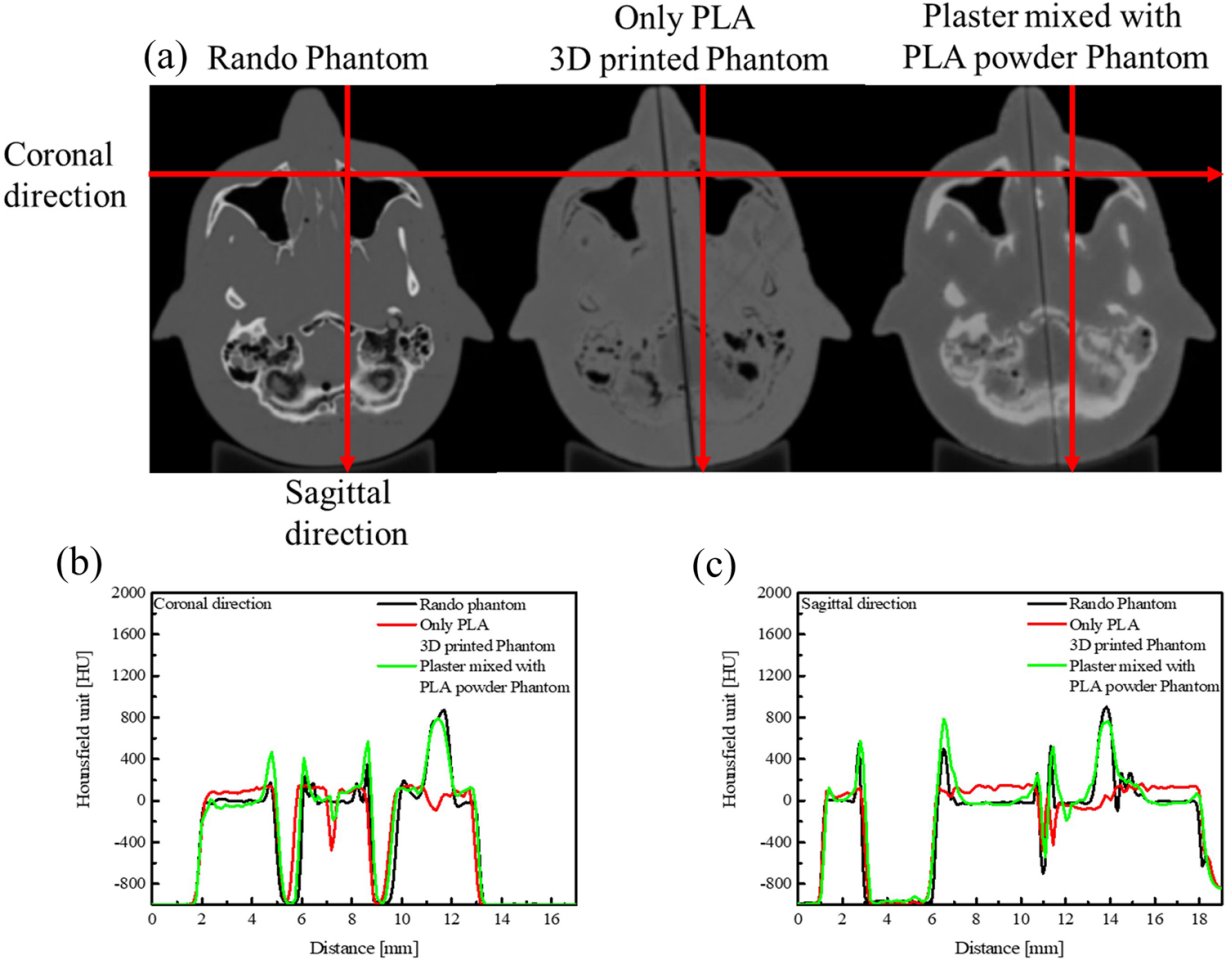
HU profiles along the red line in (**a**) the transversal plane CT images and in the (**b**) coronal and (**c**) sagittal directions.

To compare the only-PLA 3D-printed phantom and the phantom composed of plaster mixed with PLA powder, the HU profiles obtained in the same transversal plane and the same position were compared. Figure 9a depicts the transversal plane of each phantom, and Fig. 9b,c present the findings for the coronal and sagittal directions in the transversal plane. The mean difference in the soft tissue (− 500 to 200 HU) for the only-PLA 3D-printed phantom was 114 HU, and the corresponding mean difference for the phantom composed of plaster mixed with PLA powder was 61 HU, which were very similar. The mean difference in bone tissue (200 HU or more) for the only-PLA 3D-printed phantom was 544 HU, and bone tissue was not achieved. Conversely, the phantom composed of plaster mixed with PLA powder achieved a high similarity in the bone tissue, with a mean difference of 110 HU. In assessments of anatomic shape, Dice similarity coefficients (DSCs) indicate overlapping volumes between two contents; thus, the closer the DSC is to 1, the higher the matching rate, and the closer it is to 0, the lower the matching rate^16^. The DSC of the Rando phantom and the phantom composed of plaster mixed with PLA powder was evaluated in three areas (surface, bone tissue, and soft tissue), and the DSCs were 0.97, 0.73, and 0.9 for the surface, bone tissue, and soft tissue, respectively. The DSC of the bone tissue is lower than that of other regions, presumably because the plaster mixture forming the bone tissue leaked slightly into the soft tissue and air cavity regions.

## Discussion

We aimed to use a mixture of plaster powder and PLA powder to fabricate a heterogeneous phantom that can replicate the commercial Rando phantom. A 3D printer using PLA with HU values of − 884 to 169 HU obtained by adjusting the infill value (5–100%) can express the lung, fat, and soft issue. The ratio (35–0%) of the plaster to the PLA powder can be adjusted to yield HU values from 949 to 447 after drying, and the bone tissue can be expressed accordingly. The phantom composed of plaster mixed with PLA powder was produced with an infill value of 82% for soft issue and 15% PLA powder for bone tissue. In comparison with the Rando phantom, this phantom showed a difference of 15 HU for soft tissue and 53 HU for bone tissue. The DSCs of the surface, bone tissue, and soft tissue were 0.97, 0.73, and 0.9, indicating a very high level of similarity. It is thought that uniformity can be evaluated by standard deviation (SD) of the HU. In the case of Rando phantom, the SDs of bone and soft tissue were measured in 328 and 89 HU, respectively. In the case of heterogeneous phantom, the SDs of bone and soft tissue were measured in 228 and 107 HU, respectively. Comparing the SDs of these two phantoms, it is thought that they have sufficiently similar uniformity.

Besides, the mixture ratios of plaster and PLA powder showed that the HU value decreased with increasing time. If we look at Figs. 5 and 6, it is thought that these results are more likely to have changed the HU value due to evaporation of water rather than deposition.

In a similar study, Yea et al.^1^ fabricated an anthropomorphic head phantom for patient-specific QA by using a 3D printer with the FDM method. The mean HU value of the head phantom made of only one material (ABS) was − 339 HU. In Yea et al.^1^ study, nine-field IMRT was performed using 6 MV; dose measurements were evaluated by gamma index (3%/3 mm) using I'mRT MatriXX and Gafchromic EBT2 film and reported as 97.28% and 95.97%, respectively. They concluded that patient-specific IMRT QA can be performed using an anthropomorphic head phantom printed with an ABS material. However, since the anthropomorphic head phantom was fabricated using only ABS, it could not realize the HU values of various human tissues such as bone tissue. Therefore, we used plaster and PLA powder to achieve the HU value of bone tissue and improved the expressible HU value of soft tissue by using PLA of higher density.

In the studies by Kadoya et al. and Ali et al.^10,11^, the bone tissue was created using pouring techniques. Kadoya et al.^10^ used PLA filament for soft tissue and plaster for bone tissue. The water: plaster ratio in the bone tissue was 2:1. In the referenced patient CT image, the mean HU values of bone and soft tissue were reported as 12.1 and 771.5 HU, respectively. In the 3D-printed phantom, the mean HU values of bone and soft tissue were reported to be 13 and 439.5 HU. In particular, the difference in the mean HU value for bone tissue was reported to be approximately 332 HU. The DSCs of surface, bone, and soft tissue were 0.92, 0.71, and 0.81, respectively. In our data, the difference in mean HU values was approximately 51 HU, and the DSCs of surface, bone, and soft tissue were 0.97, 0.73, and 0.9, respectively. In the study by Ali et al.^11^, soft tissue was fabricated using polymethyl methacrylate (PMMA), and bone tissue was fabricated using plaster. The ratio of water to plaster powder was modified to adjust to the appropriate HU value of bone tissue. However, a higher water ratio increases the likelihood of leaks in the air cavity region of the complex structure. Therefore, we considered it more appropriate to adjust the HU value using PLA powder after selecting the appropriate water ratio.

A limitation of this study is the possibility of leakage from a complex structural area to an air cavity area, as can be seen during mixing of plaster with the PLA powder phantom in Fig. 9a. The reason is that PLA powder and plaster are in liquid form during fabricating of the bone tissue. In future studies, we plan to conduct research on dose dosimetry such as PDD and dose profile for each infill value and percentage of PLA powder. Furthermore, it is necessary to study the differences in dose distribution of complex planes such as in IMRT or VMAT depending on the presence or absence of HU implementation in the bone area.

## Conclusions

In this study, the commercial Rando phantom was replicated with appropriate HU values for bone and soft tissue. For bone tissue, the ratio of PLA powder and plaster was adjusted, and for soft tissue, the infill value of the 3D printer was adjusted. A phantom composed of a mixture of plaster and PLA powder with appropriate HU values was fabricated. This combined PLA powder and plaster replication of both soft and bone tissues of the Rando phantom was used to create a more suitable custom phantom. The fabricated phantom could thus be produced with great similarity to the commercial Rando phantom and is suitable for use in phantom-based patent-specific QA.

## Data Availability

The datasets used and/or analyzed during the current study available from the corresponding author on reasonable request.
